# Bacterial Communities in Riparian Sediments: A Large-Scale Longitudinal Distribution Pattern and Response to Dam Construction

**DOI:** 10.3389/fmicb.2018.00999

**Published:** 2018-05-16

**Authors:** Juan Chen, Peifang Wang, Chao Wang, Xun Wang, Lingzhan Miao, Sheng Liu, Qiusheng Yuan

**Affiliations:** Key Laboratory of Integrated Regulation and Resource Department on Shallow Lakes, Ministry of Education, College of Environment, Hohai University, Nanjing, China

**Keywords:** bacterial diversity, biogeographic pattern, community composition, environmental heterogeneity, hydropower dam, riparian zone

## Abstract

Sediment microbes play major roles in riparian ecosystems; however, little is known about their longitudinal distribution pattern and their responses to dam construction, the most severe human disturbance in river basins. Here, we investigated the variability of sediment bacterial communities along a large-scale longitudinal gradient and between dam-controlled and dam-affected sites in riparian zone of the Lancang River, China. The abundance, activity and diversity of sediment bacteria gradually increased in a downstream direction, but were significantly lower in the dam-affected sites than in the dam-controlled sites. The bacterial community compositions differed significantly between the upper-middle-reach and downstream sites at all control sites, and also between the dam-affected and dam-controlled sites. In the cascade dam area, the relative importance of spatial distance and environmental heterogeneity for bacterial distribution differed between the dam-controlled and dam-affected sites. Spatial distance was the primary cause of variations in bacterial community in dam-controlled site. By contrast, the environmental heterogeneity had more control over the bacterial communities than did the spatial distance in dam-affected site. Network analysis showed that the bacterial community in the dam-affected sites had lower connectivity and stability when compared with that in dam-controlled sites. These results suggest the distinct variations in sediment bacterial community in dam-affected sites, which could enhance our understanding of potential ecological effects caused by dam construction.

## Introduction

Riparian zones, at the aquatic-terrestrial interface, play an important role in protecting the land from rivers and have abundant species diversity, unique ecological communities, and high primary productivity (Huang et al., 2016). Riparian zones are extremely sensitive to changes in environmental conditions, including human disturbance and natural disasters (Huang et al., 2016). It has been suggested that sediment microbes in riparian zones can indicate the health of river basin ecosystems, because of their important roles in energy conversion, pollutant degradation, nutrient biogeochemical cycling, and element transformation and migration(Liu et al., 2011; Wu et al., 2013). Despite their importance, we have little information about the diversity and composition of microbial communities in riparian sediments because of the limits of traditional technologies. However, because of the rapid development in next-generation sequencing technology in recent years, comprehensive biogeographic surveys of microbial communities are now possible over large geographic areas (Liu et al., 2011). To date, small-scale studies (<300 km of the river) or studies based on only a few sites have been carried out (Hu et al., 2014; Kim et al., 2016); yet there is very little information about the factors that influence biogeographic patterns of bacterial communities in riparian sediments over large spatial areas.

As proposed by Baas Becking (1934), who claimed in his classic hypothesis that “everything is everywhere, but the environmental selects,” contemporary environmental factors, especially sediment properties, were initially thought to be the main controls on microbial communities (Griffiths et al., 2011; Wu et al., 2013). It is now clear that sediment pH is correlated with the spatial distribution of microbial communities in many ecosystems, including grassland (Will et al., 2010), forest (Nacke et al., 2011), tundra (Shen et al., 2013), and cropland (Fan et al., 2017). Sediment moisture (Guo et al., 2015), temperature (Zhou et al., 2016), nutrient availability (Hansel et al., 2008) and other sediment properties can also influence the composition of microbial communities. Many recent studies have shown that the distribution of sediment microbial communities was mainly affected by geographic factors such as longitude, latitude, and elevation because of microbial drift or limited dispersal over spatial distances (Schauer et al., 2010; Xiong et al., 2012; Chen et al., 2017). Martiny et al. (2006) reported that the relative importance of environmental heterogeneity and spatial distance on microbial biogeography was scale-dependent. Spatial distance is generally the main driver of variations in microbial diversity at the regional scale (>1000 km), and environmental disturbances drive variations at the local scale (Wu et al., 2013). However, we are still not sure whether spatial distance or contemporary environmental disturbances have more control on the structure of bacterial communities in a large-scale river habitat.

Dams, especially large dams or cascade dams, constructed in river systems for hydropower or water supply, are considered the most severe human disturbance on the integrity of watersheds and river ecosystems (Zhao et al., 2012; Yan et al., 2015). More than half of the world’s large river systems are affected by damming (Zhao et al., 2012). Dams significantly modify the water discharge, regulate the flow circulation, and disturb nutrient transport by retaining suspended material in reservoirs, thereby creating environments that have been subject to significant physical, chemical, and biological alterations (Li et al., 2012; Yan et al., 2015). The effects of dam construction on riparian biota and their ecological functions have attracted considerable attention in recent years (Li et al., 2018; Liu et al., 2015). Numerous studies of riparian vegetation have demonstrated that dam construction can lead to decreased habitat heterogeneity and species richness in the impounded region (Read et al., 2015), and thus greatly impacting on species’ spatial distribution (Gladyshev et al., 2015). However, there is very little information about how microbial assemblages in riparian sediments respond to river damming. A few studies have reported that the community structures of bacterioplankton suffered significant impacts from large impoundments in rivers and were correlated strongly with the variations in the physicochemical properties of water bodies (Kummu and Varis, 2007; Yan et al., 2015). Other studies found that there were clear differences in the physicochemical properties of riparian sediments before and after damming because of frequent flooding and seston sedimentation (Li et al., 2012, 2018). Consequently, we should also expect to observe changes in sediments bacterial communities.

The Lancang-Mekong River, the largest river in Asia, flows across seven climatic zones and has very diverse riparian habitats along its course from the source to the sea (Li et al., 2012; Zhao et al., 2012). There are plans to construct a cascade of 23 hydroelectric dams along the main channel of the Upper Langcang-Mekong River (i.e., Lancang River). Of these 23 dams, 6 have already been constructed in the middle and lower reaches of the river in Yunnan Province, China (Li et al., 2012; Liu et al., 2015). While the effects of dam construction on the downstream hydrological regime, water quality, and the sediment trapping efficiency have already been studied in the Lancang River (Kummu and Varis, 2007; Zhao et al., 2012; Liu et al., 2015), there is very little information about the biogeographic distribution of bacterial communities in riparian sediments along this large river and how they have changed in response to construction of the dams. The objectives of the present study were therefore to (i) investigate the longitudinal distribution of bacterial communities in riparian sediments along the Lancang River at the large scale (>1000 km), (ii) analyze the changes in the activity, abundance, diversity and composition of sediment bacterial communities in response to construction of the dams, and (iii) explore the key controls (either spatial distance or environmental heterogeneity) on the distribution of, and dam-induced variations in, bacterial communities.

## Materials and Methods

### Study Site and Sediment Sampling

**FIGURE 1:**
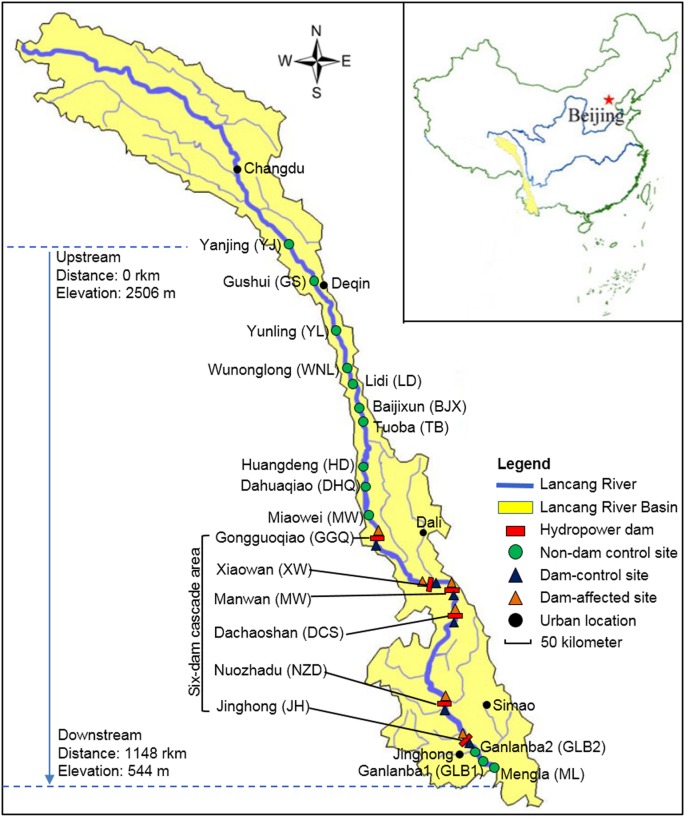
The 25 sampling sites along Lancang River, including 13 non-dam controlled sites, 6 dam-controlled sites, and 6 dam-affected sites.

The Lancang-Mekong River has its source in the Guyong-Pudigao Creek close to the foot of Mount Jifu on the Qinghai-Tibetan Plateau and discharges into the South China Sea. Passing through six countries including China, Myanmar, Laos, Thailand, Cambodia, and Vietnam, it is one of the most famous transboundary rivers in the world. The Upper Langcang-Mekong River is known as the Lancang River as it flows across China through, from upstream to downstream, Qinghai Province, Tibet Autonomous Region, and Yunnan Province. The main channel of the Lancang River, from the Jiuzhou Gauging Station to the Chinese/Myanmar border, is 2160 km long and spans an elevation of 4580 m as it flows through narrow and deep valleys. The six hydropower dams on the main channel of the Lancang River those have already been constructed in the middle and lower reaches in Yunnan Province are Gongguoqiao (GGQ), Xiaowan (XW), Manwan (MW), Dachaoshan (DCS), Nuozhadu (NZD), and Jinghong (JH) dams. Detailed information about these six dams is shown in Supplementary Table S1. A total of 25 sampling sites in the riparian zone along the mainstream of the Lancang River that spanned a distance of 1148 km from Yanjing (YJ) upstream to Mengla (ML) downstream were sampled in February 2017 (**Figure 1**). During sampling, the water flow was less than 0.15 m s^-1^ in the reservoir area and ranged from 0.60 to 1.20 m s^-1^ in the river without impoundments. We divided these sampling sites into three groups. The first group comprised control sites where there are presently no dams (non-dam control). Ten of these, including YJ, Gushui (GS), Yunling (YL), Wunonglong (WNL), Lidi (LD), Baijixun (BJX), Tuoba (TB), Huangdeng (HD), Dahuaqiao (DHQ), Miaowei (Miaowei), were upstream sites and three were downstream sites, namely Ganlanba1 (GLB1), Ganlanba2 (GLB2), and ML. The second group was dam-influenced sites that were in the reservoir area where the water level fluctuated (dam-affected). Four of these, namely GGQ-E, XW-E, MW-E, and DCS-E, were middle-reach sites, and two were downstream sites, namely NZD-E and JH-E. The third group was dam-controlled sites that were below the dam and within 5 km of the dam-affected site (dam-control). Each pair of dam-controlled site and dam-affected site was in the same climatic zone. Four of these were middle-reach sites, namely GGQ-C, XW-C, MW-C, DCS-C, and two were downstream sites, namely NZD-C and JH-C. Of the 25 sampling sites, 19 control sites that included 13 non-dam controlled sites and 6 dam-control sites were considered undisturbed and natural. All sampling sites were located near the river (within 2 m from the river water body). At each site, three replicate sediment samples were collected to a depth of 15 cm below the surface along a 10-m longitudinal transect of the river. Each individual sample was a mixture of five sediment samples that were collected randomly within a given plot with an area of 1 m^2^ (Yang et al., 2017). A total of 75 sediment samples were collected and analyzed for chemical and DNA properties.

### Chemical Property and Microbial Activity Analysis

Sediment moisture was measured gravimetrically after desiccation for 72 h at 105°C. The pH and electrical conductivity (Ec) were determined on fresh sediment at a distilled to deionized water (ddH_2_O) ratio of 1:5 (w/v) using a digital pH/Ec meter (Thermo Electron Corporation, Beverly, MA, United States). Total carbon (TC) and organic carbon (TOC) content were determined by a total organic carbon analyzer (Elementar Liqui TOC II, Frankfurt, Germany). The Kjeldahl digestion method was used to determine the total nitrogen (TN) concentrations in sediment. Total phosphorus (TP) was determined by molybdenum-blue colorimetry after digestion by hydrofluoric acid and perchloric acid. Microbial respiration rate was evaluated by the CO_2_ released per unit of time from microorganisms following the methods previously described (Hu et al., 2012). Dehydrogenase activity was determined by reduction of 2,3,5-triphenyl tetrazolium chloride to 1,3,5-triphenyl formazan (TPF) according to Tu et al. (2011).

### DNA Extraction and Quantitative Real-Time PCR (qPCR)

Genomic DNA was extracted from 0.5 g sediment using a FastDNA SPIN Kit for soil (MP Biomedicals, CA, United States) following the manufacturer’s instructions. We determined the 16S *rRNA* gene copies of total bacteria in triplicate on a real-time PCR system (StepOne Plus, Applied Biosystems, Darmstadt, Germany) using the SYBR green qPCR method with the primers P338-358F (5′-ACTCCTACGGGAGGCAGCAG-3′) and P534-518R (5′-ATTACCGCGGCTGCTGG-3′) (Yong et al., 2011). The 20 μL PCR mixture was composed of 10 μL of Master Mix (Roche Molecular Biochemicals, Mannheim, Germany), 1 μL of DNA template, 0.8 μL of each primer (20 μM), and 8.4 μL of sterile ddH_2_O. The amplification temperature program and standard curve creation were previously described (Chen et al., 2018).

### High Throughput Sequencing

The prokaryotic universal primers F515 (5′-GTGCCAGCMGCCGCGGTAA-3′) and R806 (5′-GGACTACHVGGGTWTCTAAT-3′) (Antoniou et al., 2015), with a sample-specific 12 bp barcode added to F515, were used for amplifying the V4 hypervariable region of bacterial 16S *rRNA*. The PCR reaction mixture and amplification conditions have been described elsewhere (Chen et al., 2017). Each DNA sample was amplified in triplicate and PCR products from each sample were pooled together and purified using a QIAquick PCR Purification kit (QIAGEN, CA, United States), combined in equimolar ratios in a single tube and sequenced on an Illumina HiSeq PE250 platform. The sequencing data were submitted to NCBI Sequence Read Archive database under accession number SRP125317.

### Bioinformatical and Statistical Analysis

Raw sequence data were analyzed using the Quantitative Insights Into Microbial Ecology (QIIME, version 1.8.0) pipeline (Caporaso et al., 2010). Reads with quality scores below 25 or with lengths shorter than 200 bp were removed (Huse et al., 2007). The quality sequences were clustered into operational taxonomic units (OTUs) with UCLUST based on a 97% similarity threshold using the USEARCH algorithm (Edgar, 2010). All singleton OTUs were removed during the USEARCH clustering process, because, as some singletons represent artifacts or contaminants, they would have inflated the alpha diversity erroneously (Kunin et al., 2010). The most abundant sequence from each OTU was selected as the representative sequence and the taxonomic identity of each phylotype was predicted based on the similarity to the Greengenes database^1^.

To analyze the alpha and beta diversity of sediment bacteria at the same sequencing depth, the data set was subsampled to 27 285 sequences per sample (Yang et al., 2017). The bacterial richness was calculated as the observed species in each sample and phylogenetic diversity was estimated using Faith’s index. The correlations between the alpha diversities and sediment geochemical properties were tested with linear regression analyses using SPSS 20.0. The 75 sediment samples were analyzed with non-metric multidimensional scaling analysis (NMDS) and hierarchical cluster analysis based on the Bray–Curtis distance to show the dissimilarity of sediment bacteria among the samples (beta diversity). The Mantel test was calculated as described by Chu et al. (2016) to identify which sediment geochemical properties were significantly correlated with bacterial communities, and the results were input into canonical correspondence analysis (CCA) to visualize the relationships between the sediment geochemical properties and bacterial communities. Distance–decay curves were plotted for the bacterial community similarity and spatial distance or for the bacterial community similarity and environmental distance and the slopes of the linear regression analysis (using SPSS 20.0) were used as the rates. The spatial distance was calculated using a matrix of pairwise distance along the river of all the sampling sites. The bacterial compositional similarity was calculated using the Bray–Curtis distance and the environmental distance was calculated using 10 sediment chemical properties from each sediment sample (Supplementary Table S2) based on the Euclidean distance in R. The bacterial community similarity, spatial distance, and environmental distance matrixes were linearized using PASSAGE2^2^. To compare the relative impacts of spatial distance and environmental factors on the bacterial community, a partial Mantel test was conducted based on the matrices of bacterial community similarity, spatial distance, and environmental distance. Similarity percentage (SIMPER) analysis was used to identify which OTUs contributed most to the overall dissimilarity in the bacterial community compositions between dam-controlled and dam-affected sediments. The NMDS, Mantel test, CCA, ANOSIM, SIMPER, and partial Mantel test were conducted in R with the vegan package. Heat map analysis was performed using the package heatmap in R. To explore the effect of dam construction on the co-associated properties of bacterial species, we used SpiecEasi to carry out network analysis (Cardona et al., 2016) in R and the properties were calculated in the igraph package. The topological features have been described elsewhere (Fan et al., 2017). One-way ANOVA, followed by Tukey’s test or a student’s *t*-test, was conducted using SPSS 20.0 to determine if there were any significant differences in bacterial diversity and composition similarity.

## Results

### Physiochemical Properties of Sediment Samples

Of all the measured physiochemical properties, sediment pH varied from 6.29 to 8.91, the TN concentration varied from 515 to 2719 mg kg^-1^ dry weight (dw), and the C:N ratio varied from 0.92 to 40.1. Sediment pH and the C:N ratio exhibited a gradually decreasing trend along the river, and the concentration of TN and NO_3_^-^ trended to increase from the upstream to the downstream. In the six-dam cascade area, the sediment pH in the dam-affected site was lower than that in the dam-controlled site. While, the sediment moisture, the concentration of TN, TP, and NO_3_^-^ were significantly higher in the dam-affected site than that in the dam-controlled site (Supplementary Table S2).

### Abundance and Activity of Sediment Bacteria

**FIGURE 2:**
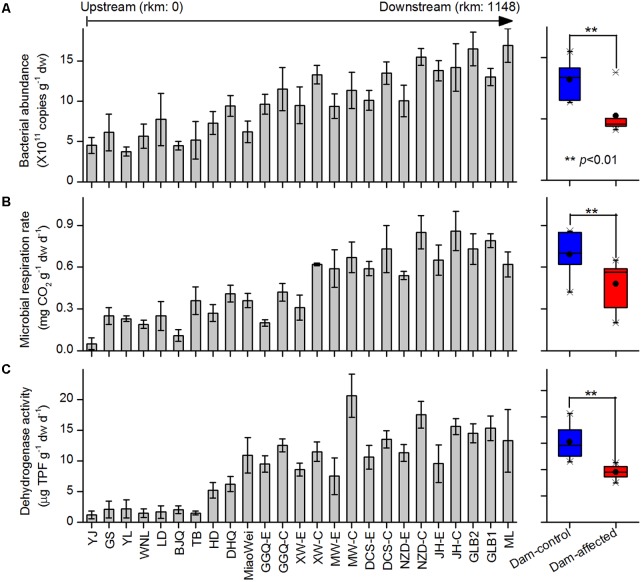
Bacterial abundance **(A)**, microbial respiration rate **(B)**, and dehydrogenase activity **(C)** in the sediments from the 25 sampling sites along the Lancang River from upstream to downstream (boxplot on the right shown the difference between the dam-control and dam-affected sites; a straight line inside the box indicates the median of the values, while a solid circle indicates the mean; the box indicates the 25 and 75% percentiles of the values; the whiskers indicate the maximum and minimum of the values).

The 16S *rRNA* gene copies quantified by qPCR were used to demonstrate the total bacterial abundance in sediment samples. Of the 25 sampling sites from upstream to downstream along the river, the 16S *rRNA* gene copies in the sediments varied between 5.30 × 10^11^ and 15.2 × 10^11^ copies g^-1^ dw, and exhibited a increasing trend in the downstream direction (**Figure 2A**). In the six-dam cascade area, the mean 16S *rRNA* gene copies of six dam-affected sites were significantly lower than that of six dam-controlled sites (**Figure 2A**). Microbial respiration rate and dehydrogenase activity were measured to indicate the microbial activities in sediment samples. Similar to the total bacterial abundance, for either microbial respiration rate or dehydrogenase activity, an increased trend was observed from the upstream sites to the downstream sites along the river, and the mean value was significantly lower in dam-affected sites than dam-controlled sites (**Figures 2B,C**).

### Diversity and Distribution of Bacterial Communities

**FIGURE 3:**
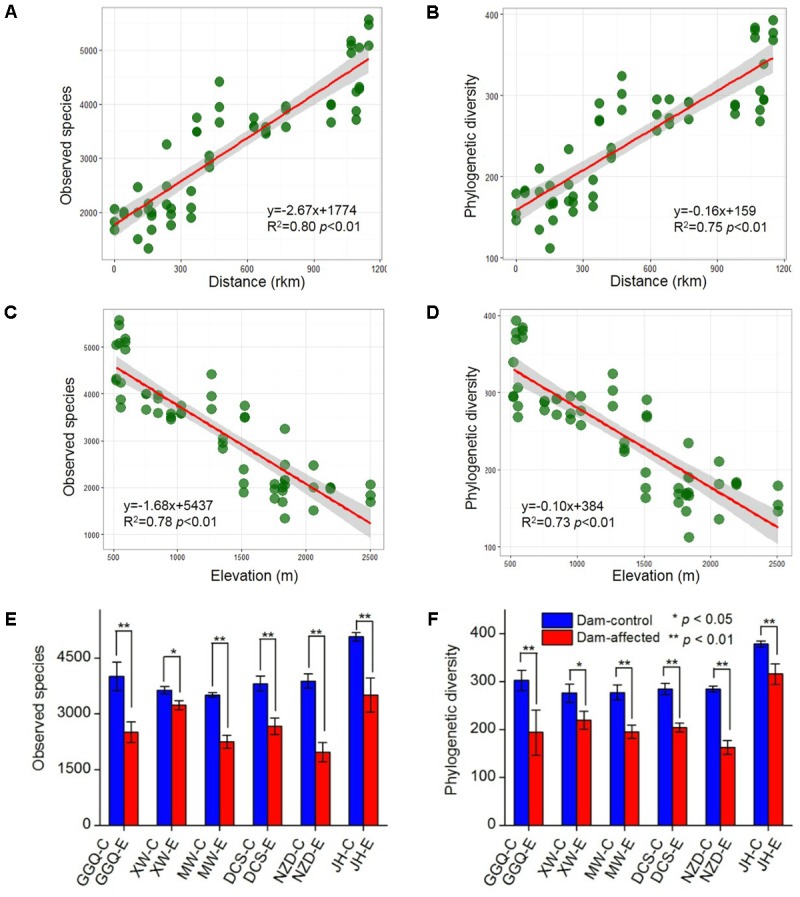
Correlation of bacterial alpha diversity with distance **(A,B)** and elevation **(C,D)** across all control sites and differences in alpha diversity between dam-controlled and dam-affected sites **(E,F)**. The shaded areas show the 95% confidence interval of the fit; ^∗^ and ^∗∗^ represent significant differences between dam-controlled and dam-affected sites when *p* ≤ 0.05 and *p* ≤ 0.01, respectively, according to Student’s *t*-test.

A total of 5 909 833 high-quality 16S *rRNA* gene V4 sequences, obtained from the 75 sediment samples collected at the 25 sites, were clustered into 40 343 bacterial OTUs based on a similarity level of 97%. Across the 19 control sites, which included 13 non-dam controlled and 6 dam-controlled sites, the bacterial alpha diversity of either observed species or polygenetic diversity tended to increase from the upstream sites to the downstream sites along the river (Supplementary Figure S1), and, as shown by linear regression analysis (**Figures 3A,B**), was significantly and positively correlated with the distance to site YJ (at km 0). The alpha diversities were significantly lower at the six dam-affected sites in the area of the six-dam cascade than at their corresponding control sites (**Figures 3E,F**).

**FIGURE 4:**
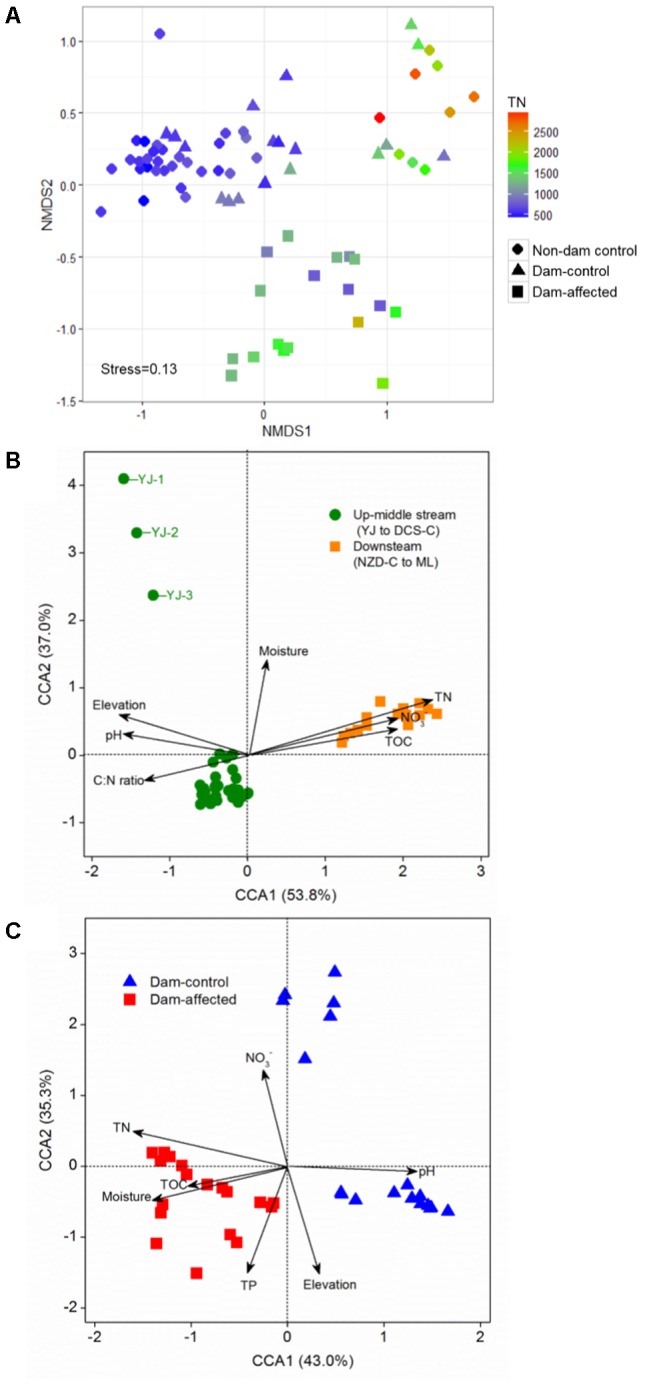
Bacterial community compositions of all 75 sediment samples as indicated by non-metric multidimensional scaling plots (NMDS) **(A)** and canonical correspondence analysis (CCA) of the geochemical factors and bacterial communities in the samples from all the control sites **(B)** and the six-dam cascade area sites **(C)**. Sampling plots in **(A)** have been color-coded according to the sediment total nitrogen (TN) content.

The NMDS showed that the samples from all the control sites, including non-dam controlled and the dam-controlled sites, formed two clusters, that is, one containing downstream sites (the sites from NZD-C to ML) and the other one containing upper-middle-reach sites (the sites from YJ to DCS-C). Samples from the dam-controlled and dam-affected sites were grouped separately by NMDS analysis, which shows that they had different bacterial community compositions (**Figure 4A**). Similarity, hierarchical cluster analysis showed that the upper-middle-reach control sites (sites from YJ to DCS-C) were clustered together and then with the dam-affected sites, which were clearly separated from the downstream control sites (sites from NZD-C to ML) (Supplementary Figure S2). Analysis by ANOSIM further confirmed that the sediment bacterial communities differed significantly between the upper-middle-reach sites and the downstream sites (*r* = 0.93, *p* < 0.001), and also between the dam-controlled sites and the dam-affected sites (*r* = 0.59, *p* < 0.001).

### Relationships Between the Bacterial Communities and Sediment Geochemical Properties

Pearson analysis showed that the bacterial alpha diversity across all the control sites was significantly correlated with sediment moisture, pH, TC, TN, the C:N ratio, and NO_3_^-^, and that there were strong negative correlations between elevation and the observed species and the polygenetic diversity (**Figure 3** and Supplementary Table S3). Significantly positive correlations were found between bacterial alpha diversity, bacterial abundance and activity (Supplementary Table S3). To identify the factors that influenced the variation in the bacterial communities among all the control sites and among the six-dam cascade area sites, the samples from all the control sites (non-dam controlled and dam controlled sites were considered together) and from the six-dam cascade area (dam-controlled and dam-affected sites were considered together) were analyzed separately using the Mantel test and CCA. The Mantel test showed that sediment TN showed the highest correlation with the community composition, both for all the control sites (*r* = 0.62, *p* = 0.001) and the six-dam cascade area (*r* = 0.32, *p* = 0.001) (**Table 1**), while NMDS results showed that the bacterial community was distributed along a TN gradient (**Figure 4A**). Elevation and some environmental variables such as TOC, NO_3_^-^, moisture, and pH were also significantly correlated with the compositions of bacterial communities in both control sites and six-dam cascade area sites (**Table 1**). The factors that significantly correlated with the bacterial community composition were selected to perform CCA, which showed that sediment TN with the longest arrow have strongest effects on bacterial community composition in either control sites or six-dam cascade area sites (**Figures 4B,C**). For the control sites, sediment TN, NO_3_^-^, TOC, pH, and elevation were important for separating the downstream sites from the upper-middle-reach sites (**Figure 4B**). In the six-dam cascade area, sediment TN, TP, moisture, and TOC contributed positively, while sediment pH contributed negatively, to separating the dam-affected sites from the dam-controlled sites (**Figure 4C**).

**Table 1:** The correlations (*r*) and significance (*p*) were determined by Mantel tests between geochemical variables and bacterial community composition in the sediments from all sampling sites, control sites and six-dam area sites (Ec, electrical conductivity; TOC, total organic carbon; TC, total carbon; TP, total phosphorus; TN, total nitrogen; NO_3_^-^, nitrate; NH_4_^+^, ammonium; ^∗∗^ represents significant correlations at *p* < 0.01).

Variable	All control sites (non-dam control and dam-control sites)		Six-dam cascade area sites (dam-control and dam-affected sites)	
	*r*	*p*	*r*	*p*
Moisture	0.21**	0.001	0.25**	0.001
pH	0.47**	0.001	0.27**	0.001
Ec	0.12	0.058	0.16	0.018
TC	0.09	0.034	0.13	0.022
TN	0.62**	0.001	0.32**	0.001
C:N ratio	0.24**	0.001	0.07	0.123
TP	0.12	0.075	0.17**	0.007
TOC	0.54**	0.001	0.18**	0.004
NO_3_^-^	0.61**	0.001	0.26**	0.001
NH_4_^+^	0.10	0.111	0.15	0.023
Elevation	0.55**	0.001	0.29**	0.001


### Relative Influence of Spatial Distance and Environmental Distance on Bacterial Communities

**FIGURE 5:**
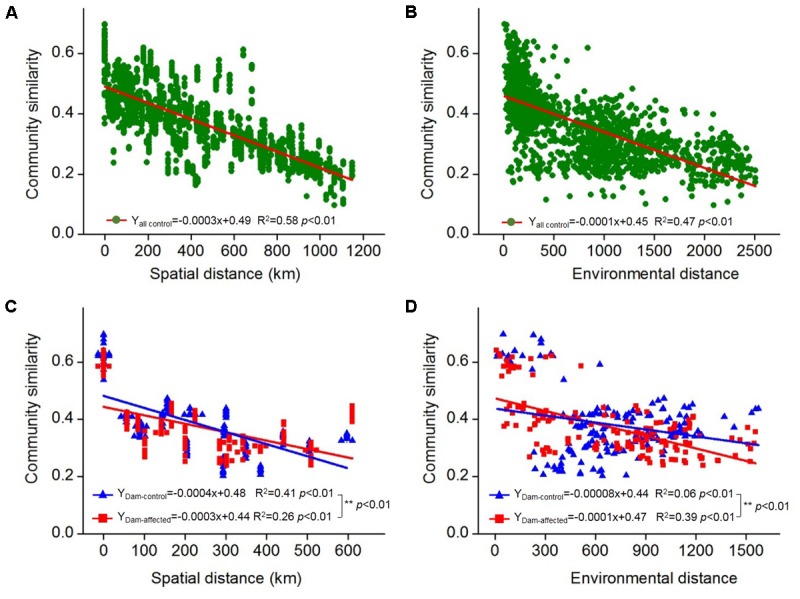
Distance–decay relationships between the bacterial community similarity, spatial distance, and environmental distance in all the control sediments **(A,B)** and the six-dam cascade area sites **(C,D)**. The slopes were compared pairwise between the dam-control and the dam-affected sediments with permutation tests and ^∗∗^ represents the significant differences when *p* ≤ 0.01.

The 25 sampling sites spanned a large spatial distance; the distance between all the control sites from YJ to ML was 1148 km; the distance between the dam-controlled sites from GGQ-C to JH-C was 597 km, while the distance between the dam-affected sites GGQ-E to JH-E was 610 km (Supplementary Table S2). Plots of community similarity versus spatial distance showed that there were distance decay relationships at all control, dam-controlled, and dam-affected sites (**Figures 5A,C**). The environmental distance was also negatively correlated with the bacterial community similarity (**Figures 5B,D**). These distance–decay curves suggest that both spatial distance and environmental factors may have influenced the distribution of the bacterial communities in sediments along the Lancang River. The spatial distance (with a higher *R*^2^ value) was more strongly correlated with the bacterial community dissimilarity than the environmental distance for all the control and dam-controlled sites. In contrast, the bacterial community composition was more strongly correlated with the environmental distance (*R*^2^ = 0.39) than with the spatial distance (*R*^2^ = 0.26) for dam-affected sites. These results were supported by the results from the partial Mantel tests, as shown in **Table 2**, which had higher *r*-values for spatial distance (0.60 and 0.64) than for environmental distance (0.44 and 0.20) for all the control and dam-controlled sites; the *r*-value for the dam-affected sites was, however, higher for environmental distance (0.55) than for spatial distance (0.39).

**Table 2:** Partial Mantel tests to compare relative impacts of spatial distance (Spa) and environmental distance (Env) on bacterial community (Spa is the geographic distance matrix and Env is the environmental heterogeneity matrix calculated with Euclidean).

Groups	Factors	*r* (correlation)	*p* (significance)
All control sites	Spa	0.60	0.001
	Env	0.44	0.001
Dam-control sites	Spa	0.64	0.001
	Env	0.20	0.015
Dam-affected sites	Spa	0.39	0.002
	Env	0.55	0.001


### Variations in the Composition of Bacterial Communities

**FIGURE 6:**
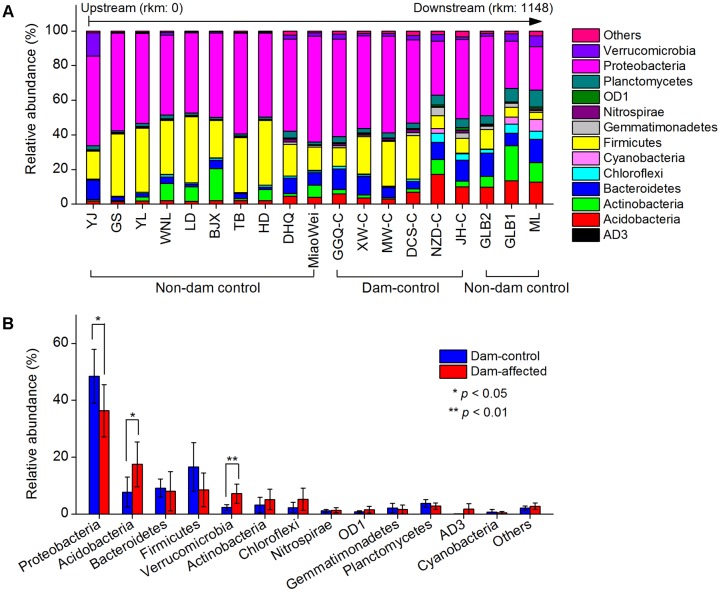
Relative abundances in the dominant bacterial phyla at all control sites along the Lancang River **(A)** and the differences in the dominant bacterial phyla between the dam-controlled and the dam-affected sediments **(B)**. Data shown in **(B)** are means of 18 replicates (6 dam-control or dam-affected sites and each one had 3 replicates). ^∗^ and ^∗∗^ represent significant differences between dam-control and dam-affected sites at *p* ≤ 0.05 and *p* ≤ 0.01, respectively, according to Student’s *t*-test.

The dominant phylum included Proteobacteria, Firmicutes, Bacteroidetes, Acidobacteria, Actinobacteria, Chloroflexi, Verrucomicrobia, and Planctomycetes, which together accounted for more than 90% of the total sequence data (**Figure 6A**). Across the 19 undisturbed natural sites, including the 13 non-dam-controlled and 6 dam-controlled sites, Acidobacteria, Bacteroidetes, and Verrucomicrobia tended to increase from the upstream sites to the downstream sites. Proteobacteria and Firmicutes were more abundant, but Chloroflexi and Planctomycetes were less abundant, at most of the upper-middle-reach sites (from YJ to DCS-C) than at the downstream sites (from NZD-C to ML). The relative abundances of the dominated phylum in the dam-controlled and dam-affected sites are shown in Supplementary Table S4. Acidobacteria, Verrucomicrobia and Chloroflexi were more abundant, and Proteobacteria and Firmicutes were less abundant, at the dam-affected sites than at their corresponding dam-controlled sites at five of the six paired dam-controlled and dam-affected sites (Supplementary Table S4). The mean relative abundances of Acidobacteria and Verrucomicrobia were significantly higher, and the mean relative abundance of Proteobacteria was significantly lower, at the dam-affected sites than at the dam-controlled sites (**Figure 6B**). As shown in Supplementary Table S5, sediment TN, the C:N ratio, TOC, and NO_3_^-^ were significantly and positively correlated with the relative abundances of Acidobacteria, Bacteroidetes, Chloroflexi, and Planctomycetes, implying that nutrients selectively enriched bacteria from these phyla. The sediment pH was negatively correlated with the relative abundance of Acidobacteria, Proteobacteria, and Firmicutes, but was positively correlated with that of Chloroflexi and Planctomycetes (Supplementary Table S5).

**FIGURE 7:**
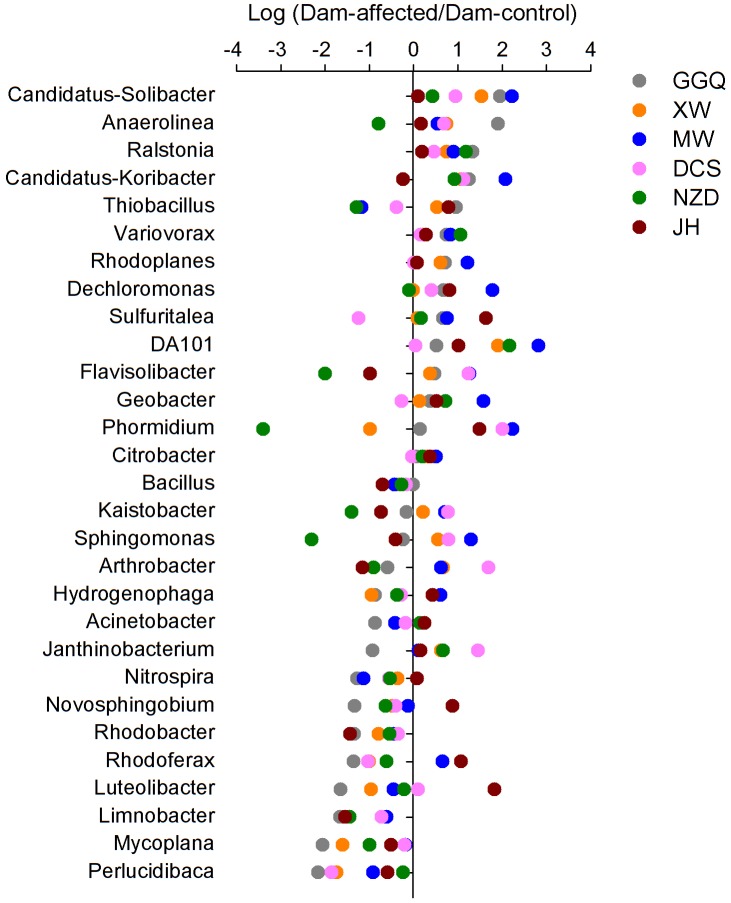
Variation in the relative abundance of the top 29 genera (relative abundance > 0.1%) between the sediments at the dam-controlled and dam-affected sites.

To visualize the effects of dam-induced water level fluctuations on the composition of bacterial communities of riparian sediments at the genus level, we calculated the logarithmic relative abundance ratios of the dam-controlled to the dam-affected sediments for the top 29 genera (that had relative abundances greater than 0.1%). Positive values represented higher abundances, and negative values represented lower abundances, at the dam-affected sites than at their corresponding dam-controlled sites. As shown in **Figure 7**, five genera, including *Candidatus Solibacter*, *Ralstonia*, *Variovorax*, *Rhodoplanes*, *DA101*, had higher relative abundances at all six dam-affected sites, and the dam-enriched *Anaerolinea*, *Candidatus-Koribacter*, *Dechloromonas*, *Sulfuritalea*, *Flavisolibacter*, and *Geobacter* were also found in at least four out of the six pairs of dam-controlled and dam-affected sites. The relative abundances of nine genera, including *Bacillus*, *Limnobacter*, *Mycoplana*, *Perlucidibaca*, *Acinetobacter*, *Rhodobacter*, *Nitrospira*, *Novosphingobium*, and *Luteolibacter*, were lower in almost all the dam-affected sites, apart from dam JH and MW. The dam-induced increases and decreases in most genera were more significant at dams MW and GGQ, respectively, than at the other dams (**Figure 7**).

**FIGURE 8:**
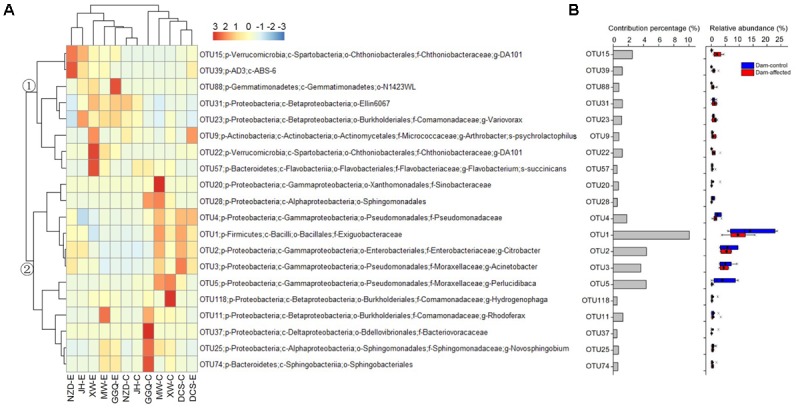
Heatmap showing the relative abundance of the top 20 OTUs with percentage contributions greater than 0.5% to generate community dissimilarity between dam-controlled and dam-affected sites **(A)** and their percentage contributions and relative abundance **(B)**. The colors represent changes in the relative abundance, and follow the color-code used in **(A)**. Two major clusters were observed, as indicated by ① and ② in the tree. In **(B)**, a straight line inside the box indicates the median of the values, while a solid circle indicates the mean; the box indicates the 25 and 75% percentiles of the values; the whiskers indicate the maximum and minimum of the values.

The results of SIMPER analysis show that, of the top 20 OTUs that accounted for more than 0.5% of the community dissimilarity, 12 OTUs belonged to the Proteobacteria phyla (**Figure 8**). With higher relative abundances, OTU1, OTU2, OTU3, OTU5, and OTU15 made greater contributions (>2% of community dissimilarity) to bacterial community dissimilarity than the other OTUs (**Figure 8B**). Based on their relative abundances, two clusters formed in the heatmap of the top 20 OTUs. Cluster 1, with 8 OTUs, had the highest abundances at most dam-affected sites, while the abundances of the 12 OTUs in cluster 2 were lower at most of the dam-affected sites than at the dam-controlled sites (**Figure 8A**). Similar results are also presented in **Figure 8B**.

### Network Topological Features of Dam-Control and Dam-Affected Sediments

**FIGURE 9:**
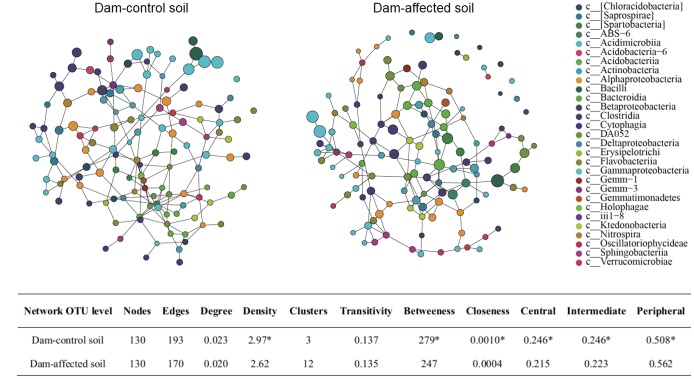
SpiecEasi network maps at the OTU level **(A)** and their network topographical features **(B)** within dam-control and dam-affected sediments. ^∗^ represents significant differences between dam-control and dam-affected sediments when *p* ≤ 0.05 according to Student’s *t*-test.

The SpiecEasi network topographical structures (**Figure 9A**) show that the dam-affected sediments had a greater number of clusters, but less density, transitivity, number of edges, degree, betweenness, and closeness than the dam-controlled sediments. The nodes were classified as peripheral, intermediate, and central according to their betweenness values for the total network. The central and intermediate nodes were less abundant, and the peripheral nodes were more abundant, in the dam-affected sediments than in the dam-controlled sediments (**Figure 9B**). Examination of the network properties of the dominant bacterial phyla showed that Firmicutes and Proteobacteria, with higher relative abundances, had very low degrees and betweenness, but that Nitrospirae, whose relative abundance was less than 2%, had the highest degree and betweenness (Supplementary Tables S4, S6). Apart from the value for Bacteroidetes, the betweenness values for all phyla were significantly lower in the dam-affected sediments than in the dam-controlled sediments (Supplementary Table S6), indicating the lower connectivity and stability of bacterial community in the dam-affected sediments.

## Discussion

### Longitudinal Distribution of Bacterial Communities in All Control Sites

A river transports surface riparian sediment, especially during the flood season, as it flows, leading to dispersal of sediment particles and microorganisms along its length (Wu et al., 2013; Staley et al., 2016). Based on the River Continuum Concept (Vannote et al., 1980), we propose that the bacteria assemblages in riparian sediments also gradually change along the river. In this study, the alpha diversity tended to increase gradually from upstream to downstream (**Figure 3** and Supplementary Figure S1), which is consistent with changes in fungal communities observed in sediment from the Changjiang River (Wu et al., 2013). However, this finding contradicts the results of previous studies in the Thames River (Gladyshev et al., 2015) and in the Danube River (Savio et al., 2015), who found that the bacterial diversity decreased from the headwaters to the river mouth. The bacterial abundance and activity gradually increased toward downstream in this study (**Figure 2**). Similarly, Ochs et al. (2010) reported that the high bacterial production in the Lower Mississippi was correlated to the increased sediment load when the large river approaches the mouth. In another study, the total bacterial number exhibited significantly increased trend along the complete length of the Danube River, but the bacterial activity did not follow a continuous trend because of the input of sewage from large cities in the midstream (Velimirov et al., 2011).

Some previous studies on large rivers, as the Danube River, have reported that the bacterial community compositions developed gradually, in general, based on the data of Joint Danube Survey 2001 and 2007 (Winter et al., 2007; Kirschner et al., 2008; Savio et al., 2015). Differently, the compositions of the bacterial communities at the upper-middle-reach sites differed significantly from those at downstream sites in this study (**Figure 4** and Supplementary Figure S2). Wu et al. (2013) also reported a similar lack of gradual change in the composition of microbial communities in sediment collected from upstream to downstream in the Changjiang River, which they attributed to uplift of the Qinghai-Tibetan Plateau. Nutrients like TN, TOC and NO_3_^-^ have been demonstrated to be important environmental variables affecting the bacterial community composition in large rivers (Kirschner et al., 2009; Gladyshev et al., 2015). In this study, the noticeably higher contents of TN, TOC, and NO_3_^-^ in the downstream (Supplementary Table S2) promoted bacterial communities that differed from those in the upper and middle reaches. The pH has also been shown to have an important role in shaping the composition of bacterial communities (Xiong et al., 2012; Shen et al., 2013). Acidobacteria, which dominates in soils with low pH values (Griffiths et al., 2011), increased as the pH decreased from upstream to downstream while, in contrast, the relative abundance of Proteobacteria decreased as the pH decreased (Supplementary Table S5). Similarly strong and significant correlations have been previously reported between the relative abundances of these two phyla and pH (Shen et al., 2013).

Even though some environmental factors were stronger predictors of community composition, we found that there were significant distance–decay relationships for bacterial community composition at both spatial and environmental distances (**Figure 5**), and that the distance decay turnover rate was greater for spatial distance than for environmental distance, as shown by the partial Mantel test (**Table 2**). This suggests that the constraints of spatial dispersal may have more control on the distribution patterns of bacteria in riparian soils from all the control sites than environmental heterogeneity, as reported in other studies (Wu et al., 2013; Chu et al., 2016; Fan et al., 2017).

### Impacts of Dam Construction on Bacterial Communities in Riparian Sediments

The construction and operation of dams can alter river hydrological regimes and cause increases in water levels in reservoir regions, which will then change the sediment physiochemical properties and influence ecological processes in riparian zones (Li et al., 2012). In this study, the changes in sediment physiochemical properties because of damming (Supplementary Table S2) may lead to the loss of some sensitive species and the enrichment of adaptable species, known as species-sorting (Ruiz-Gonzalez et al., 2013). Also, fluctuations in inundation induced by dam construction can accelerate the exchange of energy and materials between a river and its flooded area (New and Xie, 2008; Li et al., 2012), which may contribute to species dispersal along the riparian zone and increase the invasion of introduced or exotic species from upstream areas. Allochthonous inputs can be described by the so-called mass-effect, where the dispersal of organisms exceeds the rate of local extinction (Ruiz-Gonzalez et al., 2013). The decreases in bacterial diversity and abundance in dam-affected riparian areas (**Figures 2**, **3**) suggest that species-sorting has more influence than mass effects in shaping microbial communities, as also proposed by Savio et al. (2015).

We also found that the composition of bacterial communities differed significantly between the dam-affected and dam-controlled sites (**Figure 4**), as reported in previous studies (Ruiz-Gonzalez et al., 2013; Yan et al., 2015). Results from the distance decay relationship analysis and partial Mantel test confirmed that the sediment bacterial communities were mainly shaped by spatial distance in the dam-controlled sites, which was also the case for the control sites in this study (**Figure 5** and **Table 2**) and for some other natural areas, such as the Tibetan Plateau (Chu et al., 2016; Yang et al., 2017). In contrast, we found that the contribution of environmental distance became more important than that of spatial distance for the dam-affected sites, which further confirms that environmental heterogeneity induced by damming was an important influence on the composition of bacterial communities in riparian sediments. Decreases in the contribution of spatial distance, and increases in the contribution of environmental factors, to bacterial communities were also reported in previous studies of other anthropogenic activities such as fertilization (Chen et al., 2017).

The responses of the dominant bacterial phyla to dam construction, such as Acidobacteria and Verrucomicrobia, which had higher relative abundances in dam-affected sediments (**Figure 6**), might be linked to the lower sediment pH and higher sediment moisture in this area (Supplementary Table S2), respectively, as it has been reported that Acidobacteria prefers a low pH environment (Griffiths et al., 2011) and the abundance of Verrucomicrobia was positively correlated to sediment moisture (Buckley and Schmidt, 2001). At the genus level, it is noteworthy that the relative abundance of some functional bacteria, including *Nitrospira* for nitrite oxidation (Lücker and Delong, 2010), *Citrobacter* for nitrate reduction (Huang and Tseng, 2001), *Sulfuritalea* and *Limnobacter* for sulfate oxidation (Lu et al., 2011; Watanabe et al., 2014), and *Geobacter* for iron reduction (Snoeyenbos-West et al., 2000), were significantly altered, implying that dam construction may impact microbial-mediated element biogeochemical cycling in riparian zones. Importantly, the relative abundance of the genus *Ralstonia*, a devastating, soil-borne plant pathogen with a global distribution and an unusually wide host range (Salanoubat et al., 2002), increased significantly at dam-affected sites in this study (**Figure 7**), and increases of this genus might pose a threat to the riparian vegetation and health of the river basin ecosystem.

At the OTU level, several OTUs with high relative abundances contributed most to the differences in bacterial communities between the dam-controlled and dam-affected sediments (**Figure 8**), as also reported by Chu et al. (2016). Network analysis has been used in microbial ecological studies to visualize the co-associated properties of bacterial species and to predict the possible ecological processes that are regulated by bacterial communities (Fuhrman, 2009; Cardona et al., 2016). In this study, the number of no co-occurrences across different OTUs was greater, and less compact topologies with lower density and transitivity occurred more frequently, at the dam-affected sites than at the dam-controlled sites (**Figure 9**). This suggests that dam construction might obstruct the interactions and coupling among bacterial species and may also impact the assemblages and stability of communities in the riparian zone (Fuhrman, 2009). The low level of interactions in dam-affected sediment might be associated with the low bacterial diversity and the environmental filtering effect induced by damming (**Figure 3**), as reported by Ding et al. (2015). While Nitrospirae had a lower relative abundance than the other phyla, it maintained greater connectivity (Supplementary Table S6), perhaps because the bacterial species in this phyla need to cooperate with other bacteria when mediating N cycling (Fan et al., 2017). In contrast, Firmicutes, which was more abundant, had less, or even no, connectivity (Supplementary Table S6), which might be explained by their strong adaptation to riparian habitats (Johnston and Leff, 2015), such that they do not need to interact with other species.

## Conclusion

In this contribution, we have reported information about the longitudinal distribution of bacterial communities in riparian sediments along the Lancang River and their responses to dam construction. We found that the bacterial diversity increased gradually along the river from upstream to downstream and that the compositions of the communities differed significantly between the upper-middle-reach sites and the downstream sites. This biogeographic distribution might be mainly driven by the limitation of microbial dispersal or drift, which are mostly influenced by spatial distance between the sites. Dam construction significantly reduced the bacterial diversity and caused shifts in the community composition in riparian sediments. In contrast to the dam-controlled sites, environmental heterogeneity made a greater contribution to bacterial community composition than did spatial distance at dam-affected sites. The interactions among different bacteria species also decreased because of the presence of dams. This study reveals the strong impacts of dam construction on bacterial communities in riparian sediments, thus challenging the sustainability of river ecosystems with the expanding hydropower development. However, this study only focused on the general changes of total bacterial communities using 16S *rRNA* gene amplicon sequencing, a major aspect missing from this study is information on the functioning of bacterial assemblages and their potential responses to damming. More specific researches should be conducted to better understand of the functional responses of sediment bacteria in riparian zone due to dam construction.

## Author Contributions

JC, PW, and CW conceived and designed the research. JC, XW, and LM performed the research. LM, SL, and QY analyzed the data. JC, PW, and XW contributed to the writing of the manuscript.

## Conflict of Interest Statement

The authors declare that the research was conducted in the absence of any commercial or financial relationships that could be construed as a potential conflict of interest.

## Footnotes

1 http://greengenes.lbl.gov/

2 www.passagesoftware.net
