# Supplementary material for: Bacterial Communities in Riparian Sediments: A Large-Scale Longitudinal Distribution Pattern and Response to Dam Construction
Source: Front Microbiol. 2018 May 16;9:999. doi: 10.3389/fmicb.2018.00999 (PMC5964209; doi:10.3389/fmicb.2018.00999)
Supplement: Supplementary file 1 [file Presentation_1.PDF]

Supporting Materials

Title: Bacterial communities in riparian sediments: a large-scale longitudinal distribution pattern and response to dam construction

Authors: Juan Chen, Peifang Wang*, Chao Wang, Xun Wang, Lingzhan Miao, Sheng Liu, Qiusheng Yuan

Institute or laboratory of origin:

Key Laboratory of Integrated Regulation and Resource Department on Shallow Lakes, Ministry of Education, College of Environment, Hohai University, 1 Xikang Road, Nanjing 210098, P.R. China

* **Corresponding Author**

Address: College of Environment, Hohai University, Xikang Road No.1 Nanjing, 210098, Jiangsu Province, P.R. China

Telephone: +86-025-83787930; Fax: +86-025-83787332

E-mail address: pfwang2005@hhu.edu.cn

Main content:

Figs. S1 and S2, Table S1 to S6

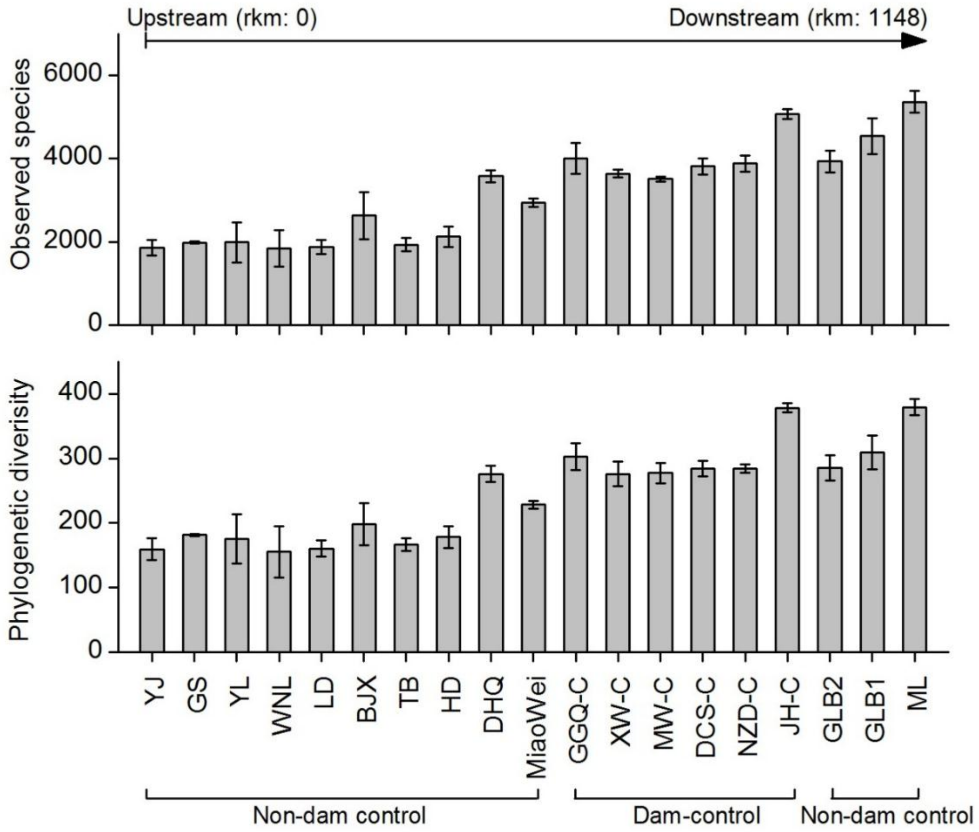

Fig. S1 Alpha diversity of observed species and phylogenetic diversity in the 19 undisturbed control sites along the Lancang River from upstream to downstream.

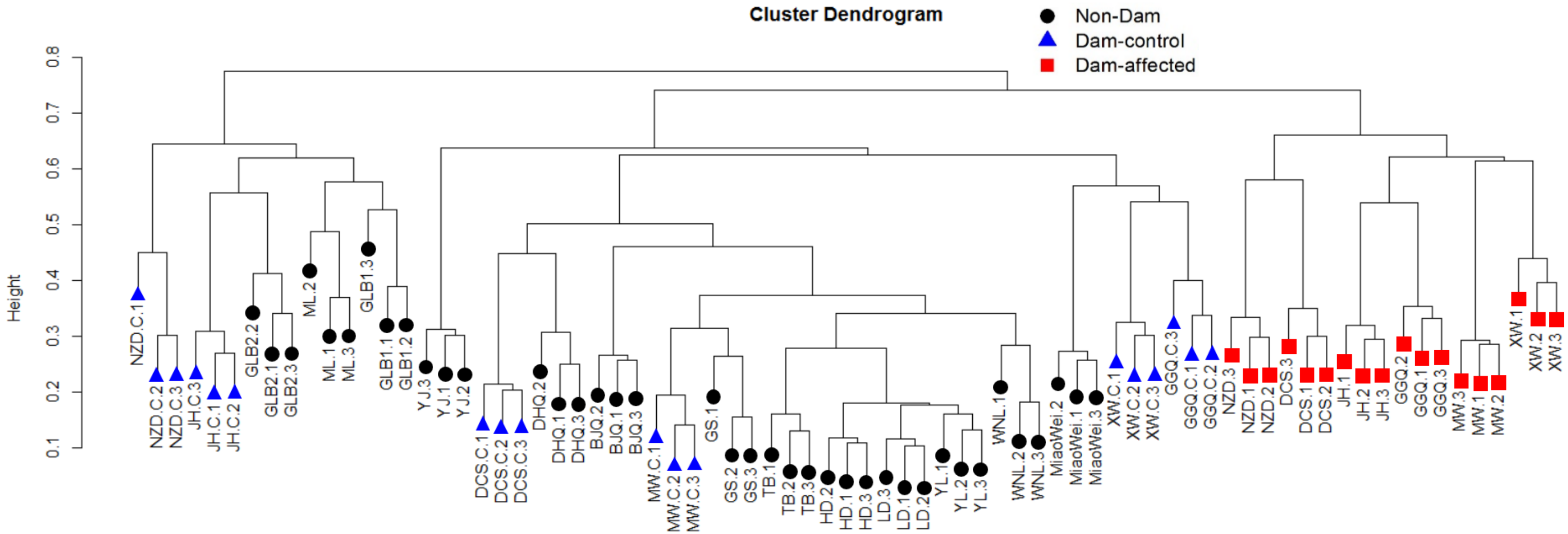

Fig. S2 Hierarchical cluster dendrogram of the bacterial community in all 75 sediment samples from 25 riparian sites along the Lancang River by Bray-Curtis distance.

Table S1. The six cascade hydropower dams constructed in the middle-lower reaches of the Lancang River.

Name	Dam			Reservoir			Installed capacity (MW)	Operation year
	Height (m)	Normal water level (m)	Location altitude (m)	Drop (m)	Volume (10^8 m^3)	Area km^2		
Gongguoqiao (GGQ)	130	1319	1242	77	5.1	16.72	750	2013
Xiaowan (XW)	292	1240	988	252	151.32	189.10	4200	2010
Manwan (MW)	132	994	895	99	10.6	23.6	1500	1995
Dachaoshan (DCS)	120	899	815	84	8.84	26.25	1350	2003
Nuozhadu (NZD)	262	812	602	210	233.68	320	5850	2017
Jinghong (JH)	107	602	535	67	12.33	32.8	1500	2009

Table S2. Description of the sediment variables by study sites (Values are the means of three replicates. Ec, electrical conductivity; TN, total nitrogen; TC, total carbon content; TOC, total organic carbon content; TP: total phosphorus, NO_3^-: nitrate, NH_4^+: ammonium).

**Group**	**Site**	**Code**	**Longitude**	**Latitude**	**Moisture (%)**	**pH**	**Ec (μm s^{-1})**	**TC (%)**	**TN (mg kg^{-1} DW)**	**C:N ratio**	**TP (mg kg^{-1} DW)**	**TOC (%)**	**NO_3^- (mg kg^{-1} DW)**	**NH_4^+ (mg kg^{-1} DW)**	**Elevation (m)**	**Distance (rkm)**
Non-dam control	Yanjing	YJ	N29°01'21"	E98°35'49"	10.5	8.91	206	0.81	573	20.2	404	0.02	0.76	1.95	2506	0
Non-dam control	Gushui	GS	N28°43'22"	E98°40'59"	4.43	8.70	51.0	0.56	628	9.17	610	0.18	0.46	1.85	2196	40
Non-dam control	Yunling	YL	N28°16'31"	E98°51'28"	0.27	8.49	75.8	1.12	524	22.4	499	0.07	0.53	1.91	2064	104
Non-dam control	Wunonglong	WNL	N27°54'12"	E98°58'20"	0.51	8.65	67.2	1.01	659	20.7	486	0.03	1.41	2.17	1840	152
Non-dam control	Lidi	LD	N27°48'48"	E99°2'21"	1.05	8.63	55.3	0.88	619	19.3	457	0.03	1.54	2.54	1820	166
Non-dam control	Baijixun	BJX	N27°19'9"	E99°6'13"	0.40	7.85	69.0	0.60	743	10.5	572	0.13	2.79	2.75	1837	235
Non-dam control	Tuoba	TB	N27°11'12"	E99°7'48"	5.40	8.22	105	0.91	515	19.5	466	0.15	1.19	1.98	1760	253
Non-dam control	Huangdeng	HD	N26°29'4"	E99°8'20"	2.11	8.30	121	1.06	634	20.7	509	0.08	1.82	2.15	1517	345
Non-dam control	Dahuaqiao	DHQ	N26°17'47"	E99°8'8"	5.31	8.57	66.7	0.85	579	16.7	509	0.05	0.65	1.99	1524	371
Non-dam control	Miaowei	MiaoWei	N25°51'59"	E99°9'27"	5.57	8.28	144	0.47	823	5.91	789	0.17	0.91	2.26	1355	426
Dam-affected site	Gongguoqiao	GGQ-E	N25°38'41"	E99°15'54"	23.0	8.50	131	1.23	781	15.7	782	0.52	1.83	2.71	1313	455
Dam-control site	Gongguoqiao	GGQ-C	N25°32'18"	E99°20'3"	5.37	7.97	56.4	0.99	663	13.7	721	0.28	1.56	2.17	1266	472
Dam-affected site	Xiaowan	XW-E	N24°43'3"	E100°5'13"	15.9	6.29	29.9	0.76	1972	7.27	1039	0.74	3.76	4.54	1201	623
Dam-control site	Xiaowan	XW-C	N24°40'4"	E100°5'47"	5.61	8.24	31.6	0.08	581	1.09	694	0.02	1.03	2.00	1031	628
Dam-affected site	Manwan	MW-E	N24°22'32"	E100°16'7"	16.0	7.12	48.4	1.13	1235	24.3	465	0.45	1.84	2.43	993	680
Dam-control site	Manwan	MW-C	N24°21'42"	E100°16'36"	10.9	8.78	18.1	0.34	615	8.02	426	0.33	1.51	2.08	950	684
Dam-affected site	Dachaoshan	DCS-E	N24°1'52"	E100°13'2"	23.4	6.81	44.4	0.29	1198	1.89	1524	0.28	1.61	2.71	932	764
Dam-control site	Dachaoshan	DCS-C	N23°35'31"	E100°13'50"	6.90	7.12	22.7	0.12	953	0.83	1505	0.12	0.46	1.01	848	773
Dam-affected site	Nuozhadu	NZD-E	N22°24'2"	E100°13'36"	11.2	6.12	15.8	0.37	1366	3.87	952	0.36	2.93	2.56	823	965
Dam-control site	Nuozhadu	NZD-C	N22°21'18"	E100°16'47"	9.03	7.32	9.27	0.38	1436	40.9	93	0.37	2.97	2.37	758	977

Dam-affected site	Jinghong	JH-E	N22 °3'33"	E100 °45'33"	23.9	7.62	39.7	0.04	1643	0.92	481	0.04	1.06	1.84	592	1065
Dam-control site	Jinghong	JH-C	N22 °3'10"	E100 °45'25"	10.5	7.58	77.7	0.29	1174	9.32	310	0.26	9.93	1.91	594	1069
Non-dam site	Ganlanba2	GLB2	N21 °53'14"	E100 °54'8"	10.5	7.58	90.1	0.50	1745	18.5	271	0.49	15.62	1.88	559	1091
Non-dam site	Ganlanba1	GLB1	N21 °51'12"	E101 °0'17"	1.87	7.53	108	0.90	2719	17.1	523	0.82	10.09	2.82	523	1106
Non-dam site	Mengla	ML	N21 °43'2"	E101 °7'17"	5.31	7.15	76.7	0.90	2334	29.1	308	0.88	11.29	2.66	544	1148

Table S3. The correlations (r) and significance (*p*) were determined by Pearson analysis between diversity and environmental variables, bacterial abundance and microbial activity across all undisturbed control sites including 13 non-dam control and 6 dam-control sites (Ec: electrical conductivity, TOC: total organic carbon, TC: total carbon, TP: total phosphorus, TN: total nitrogen, NO_3^-: nitrate, NH_4^+: ammonium; values in bold indicate significant correlations at $p < 0.01$).

	Variable	Observed species		Phylogenetic diversity	
		**r**	***p***	**r**	***p***
**Environmental variables**	**Moisture**	**0.411**	**0.002**	**0.449**	**<0.001**
	**pH**	**-0.715**	**<0.001**	**-0.672**	**<0.001**
	Ec	-0.270	0.042	-0.301	0.023
	**TC**	**-0.381**	**0.003**	**-0.404**	**0.002**
	**TN**	**0.683**	**<0.001**	**0.612**	**<0.001**
	**C:N ratio**	**-0.702**	**<0.001**	**-0.680**	**<0.001**
	TP	-0.060	0.660	-0.056	0.677
	**TOC**	**0.684**	**<0.001**	**0.634**	**<0.001**
	**NO_3^-**	**0.645**	**<0.001**	**0.603**	**<0.001**
	NH_4^+	0.118	0.383	0.053	0.697
	**Elevation**	**-0.881**	**<0.001**	**-0.856**	**<0.001**
**Bacterial abundance**	**16S *rRNA* gene copies**	**0.847**	**<0.001**	**0.874**	**<0.001**
**Microbial activity**	**Microbial respiration rate**	**0.802**	**<0.001**	**0.826**	**<0.001**
	**Dehydrogenase activity**	**0.800**	**<0.001**	**0.822**	**<0.001**

Table S4. The relative abundance (%) of dominate phylum in the sediments from dam-control and dam-affected sites. GGQ: Gongguoqiang, XW: Xiaowan, MW: Manwan, DCS: Dachaoshan, NZD: Nuozhadu, JH: Jinghong, C: dam-control site, E: dam-affected site; values are the means of three replicates.

**Phylum**	**GGQ**		**XW**		**MW**		**DCS**		**NZD**		**JH**	
	**GGQ-C**	**GGQ-E**	**XW-C**	**XW-E**	**MW-C**	**MW-E**	**DCS-C**	**DCS-E**	**NZD-C**	**NZD-E**	**JH-C**	**JH-E**
AD3	0.02	0.51	0.03	1.12	0.00	1.46	0.13	1.70	0.00	5.47	0.05	0.08
Acidobacteria	5.92	27.1	3.50	23.0	2.93	11.7	6.75	15.4	17.3	21.7	10.0	5.87
Actinobacteria	2.45	5.09	1.85	4.49	0.85	1.67	1.86	9.79	8.59	8.74	3.31	0.98
Bacteroidetes	11.9	5.25	10.6	6.96	5.88	14.1	4.29	2.90	9.93	0.68	12.0	18.5
Chloroflexi	1.48	5.41	1.07	2.04	0.54	2.15	1.45	6.61	5.25	12.2	3.77	2.85
Cyanobacteria	0.09	0.18	0.43	0.12	0.21	1.42	0.04	0.33	2.65	0.02	0.33	0.59
Firmicutes	10.7	2.71	21.5	4.72	25.9	9.83	25.1	18.5	7.46	11.1	8.50	4.26
Gemmatimonadetes	1.14	2.13	0.85	4.38	0.65	0.90	1.74	1.37	4.87	0.16	3.22	0.63
Nitrospirae	1.24	0.41	0.80	1.43	1.05	0.77	1.74	2.77	0.28	0.59	1.52	1.95
OD1	0.57	3.01	0.45	2.83	0.32	0.85	0.63	0.02	0.92	1.75	1.58	0.83
Planctomycetes	3.35	4.51	2.58	2.35	2.79	2.18	3.10	2.59	5.66	1.29	5.10	3.71
Proteobacteria	56.4	35.9	53.6	34.8	56.0	44.4	48.1	33.1	31.3	21.9	45.7	48.0
Verrucomicrobia	2.85	3.75	1.40	7.97	1.53	6.80	2.60	3.49	3.95	12.5	1.56	8.41
Others	1.86	3.98	1.37	3.82	1.41	1.87	2.49	1.46	1.93	1.95	3.36	3.38

Table S5. The Pearson's correlation analysis of relative abundance of several dominant phyla and environmental variables in all control sediments. Ec: electrical conductivity, TOC: total organic carbon, TC: total carbon, TP: total phosphorus, TN: total nitrogen, NO_3^-: nitrate, NH_4^+: ammonium; *and ** indicate significant correlations at $p < 0.05$ and $p < 0.01$, respectively.

**Phylum**	**Moisture**	**pH**	**Ec**	**TC**	**TN**	**C:N ratio**	**TP**	**TOC**	**NO_3^-**	**NH_4^+**	**Elevation**
Acidobacteria	0.356	-0.810**	-0.217	-0.228	0.832**	0.449	-0.292	0.766**	0.662**	0.235	-0.803**
Bacteroidetes	0.641**	-0.431	0.092	-0.284	0.463*	0.111	-0.294	0.474*	0.583**	0.084	-0.547*
Chloroflexi	0.148	-0.754**	-0.143	-0.083	0.865**	0.482*	-0.353	0.776**	0.655**	0.418	-0.728**
Proteobacteria	0.062	0.651**	0.117	-0.100	-0.854**	-0.586**	0.352	-0.740**	-0.616**	-0.478*	0.550*
Verrucomicrobia	0.396	-0.101	0.500*	0.078	0.336	0.308	-0.149	0.296	0.154	0.096	0.004
Firmicutes	-0.489*	0.624**	-0.100	0.253	-0.692**	-0.164	0.222	-0.671**	-0.616**	-0.280	0.667**
Planctomycetes	0.261	-0.688**	-0.084	-0.030	0.902**	0.383	-0.300	0.882**	0.741**	0.325	-0.755**

Table S6. Spiec-Easi network topological statistic coefficients of different bacteria phyla.

**Phylum**	**Network Topological Features**					
	**Degree**		**Betweenness**		**Closeness**	
	**Dam-control sediment**	**Dam-affected sediment**	**Dam-control sediment**	**Dam-affected sediment**	**Dam-control sediment**	**Dam-affected sediment**
Acidobacteria	1.6	1.2	37.2	16.6	0.0004	0.0003
Actinobacteria	1.3	1.5	15.0	0.50	0.0004	0.0003
AD3	1.5	0.5	15.5	0.00	0.0003	0.0003
Armatimonadetes	1.0	1.0	96.0	0.00	0.0003	0.0003
Bacteroidetes	1.2	2.7	38.5	52.5	0.0003	0.0003
Chlorobi	1.0	1.5	15.5	5.00	0.0003	0.0003
Chloroflexi	0.5	2.0	1.50	18.5	0.0003	0.0003
Cyanobacteria	2.0	2.0	30.5	21.5	0.0004	0.0004
Elusimicrobia	2.0	0.0	31.0	0.00	0.0004	0.0002
Firmicutes	0.3	1.0	0.00	6.00	0.0003	0.0003
GAL15	0.0	2.0	0.00	1.00	0.0002	0.0002
Gemmatimonadetes	1.2	1.0	38.2	0.00	0.0004	0.0003
Nitrospirae	5.0	3.0	312	127	0.0005	0.0004
OD1	0.0	3.0	0.00	3.00	0.0002	0.0003
Planctomycetes	1.7	0.7	109	0.00	0.0004	0.0003
Proteobacteria	1.4	1.5	42.3	16.5	0.0003	0.0003
Unclassified	0.0	1.0	0.00	0.00	0.0002	0.0002
Verrucomicrobia	2.6	1.2	66.6	27.8	0.0004	0.0003
WS3	1.0	0.0	0.00	0.00	0.0004	0.0002
